# Soil Parameters and Forest Structure Commonly Form the Microbiome Composition and Activity of Topsoil Layers in Planted Forests

**DOI:** 10.3390/microorganisms12061162

**Published:** 2024-06-06

**Authors:** Katalin Bereczki, Endre György Tóth, Tibor Szili-Kovács, Melinda Megyes, Kristóf Korponai, Botond Boldizsár Lados, Gábor Illés, Attila Benke, Károly Márialigeti

**Affiliations:** 1 Doctoral School of Environmental Sciences, Eötvös Loránd University, 1117 Budapest, Hungary; megyesmelinda@gmail.com; 2 Department of Forest Management and Ecology, Forest Research Institute, University of Sopron, 9600 Sárvár, Hungary; illes.gabor@uni-sopron.hu; 3 National Coalition of Independent Scholars (NCIS), Brattleboro, VT 05301, USA; endre.toth@ncis.org; 4 Institute for Soil Sciences, Centre for Agricultural Research, 1022 Budapest, Hungary; szili-kovacs.tibor@atk.hu; 5 Department of Plant Molecular Biology, Agricultural Institute, Centre for Agricultural Research, 2462 Martonvásár, Hungary; korponai.kristof@gmail.com; 6 Department of Forestry Breeding, Forest Research Institute, University of Sopron, 9600 Sárvár, Hungary; lados.botond@uni-sopron.hu (B.B.L.); benke.attila@uni-sopron.hu (A.B.); 7 Department of Microbiology, Eötvös Loránd University, 1117 Budapest, Hungary; marialigeti.karoly@ttk.elte.hu

**Keywords:** bacterial diversity, bacterial community structure, substrate-induced respiration, citrate, pedunculate oak, black locust

## Abstract

Soil bacterial communities play a remarkable role in nutrient cycling, significantly affecting soil organic material content, soil fertility, and, in an indirect way, plant succession processes. Conversely, vegetation type influences microbial soil life. The present study compared the bacterial microbiome composition, diversity and catabolic activity profile of topsoil samples collected under three different forest types (a twice-coppiced black locust stand, a young, naturally reforested, and a middle-aged mixed pedunculate oak stand) planted on former arable land in the early 20th century. Diversity indices determined during 16S ribosomal RNA sequencing-based metagenome analysis indicated that the black locust stand had the highest soil bacterial community diversity. At the phylum level, Acidobacteriota, Actinobacteriota, Proteobacteria, Verrucomicrobiota, Bacteroidota, and Gemmatimonadota were the most abundant taxa in the forest soils. Concerning soil parameters, redundancy analysis revealed that pH had the highest impact on bacterial community structure and pH, and soil organic carbon content on the samples’ respiration patterns. As for catabolic activity, the recently clearcut oak forest showed the lowest substrate-induced respiration, and citrate was the main driver for the inter-stand variability of microbial activity. Our results confirm that soil parameters and forest type influence the composition and functioning of the soil bacterial microbiome.

## 1. Introduction

Compared to other terrestrial ecosystems, forests play the most determinant role in atmospheric CO_2_ sequestration and carbon storage [1]. The estimated amount of carbon stored in forests exceeds 860 Pg, which is allocated primarily to soil (44%) and live biomass (42%) [2]. The carbon sequestration ability of forests supports afforestation as a major tool in climate change mitigation, despite its dependence on regional factors [3,4,5]. In addition to its global effect, afforestation could have a remarkable impact on smaller, local scales, including catchment area water yields [6,7], plant biodiversity [8,9], animal community composition [10,11], and soil physicochemical properties [12,13]. The soil property or soil quality changes after afforestation are similar to secondary forest vegetation effects [14], illustrating the significance of planted forests for soils and ecology.

The species richness of soil-related life forms is substantial, in which the microbiome, namely prokaryotes, occupies a prominent role [15]. Additionally, the estimated biomass possessed by soil microbiomes compared to other groups (e.g., soil meso-microfauna, plants) is significantly higher [16], highlighting the importance of soil microorganisms. The ecological value of soil bacteria is well reflected by their role in element cycling, such as carbon, nitrogen, metals or phosphorous, and in the decomposition of dead plant biomass [17], through which their existence and functioning are crucial in the life of forests. The role of soil bacteria in the biodegradation of pollutants is also prominent [18,19].

The compositions of forest soil bacterial communities typically differ from those of non-forest vegetation types, such as grasslands [20,21]. The change in vegetation type can lead to shifts in the composition and functioning of soil bacterial communities, as observed after afforestation activities [22,23].

Although the initial ecological factor role is determinant, differences in the structure of planted forests or plantations—such as different compositions of tree species or canopy closure—often cause alterations in the structure and activity of soil bacteria communities [24,25,26,27]. The differential effect of trees and tree density on edaphic parameters, like soil pH, moisture content, or nutrient availability, are chiefly responsible for the change, which can occur directly (root exudates) and indirectly (litter quality) [25].

Forest harvesting also influences the structure and activity of the soil bacterial community, although the direction and rate of change do not follow strict regularity. Generally, logging has a more pronounced effect on community functioning than on composition and typically induces decreased respiration activity [28,29,30]. Organic matter removal and soil compaction could be significant causes of change [31]. Therefore, forest stands afforested using different tree species and managed with varying harvesting methods in both time and frequency should display notable dissimilarities in soil bacterial microbiome composition and functioning.

We comparatively analyzed soil bacterial composition and activity in three planted forest stands in Central Hungary established on former arable land in the 1930s. The stands differed in species composition (native contra indigenous species) and were in different stages of forest use (regeneration, growing, and before the harvest period). We hypothesized that (a) the recently clear-cut oak forest has lower soil bacterial diversity and activity than the older forests due to the detrimental effects of harvesting; (b) in the two mature stands, the oak forest possesses higher soil bacterial diversity than the twice-harvested non-indigenous tree plantation; (c) because black locust is a nitrogen-fixing plant, soil nitrogen is a principal driver of inter-stand variability of soil bacterial communities. To perform the investigations, we collected soil samples randomly in 100 m^2^ experimental parcels placed on a typical and homogenous part of the stands, ensuring minimal disturbance in the forests. Bacterial diversity and taxa relative abundance data were derived from 16S RNA sequence data. For microbial activity analyses, we used a substrate-induced respiration method.

## 2. Materials and Methods

### 2.1. Description of Study Sites

**Figure 1:**
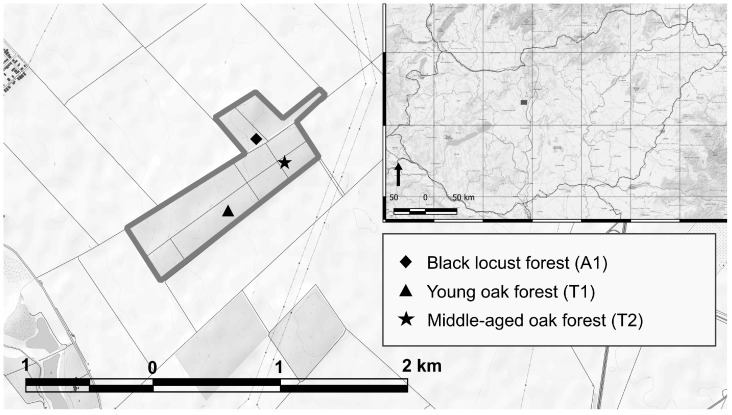
Locations of sampling sites at the border of Martonvásár and Ráckeresztúr, Hungary; map sources: OpenTopoMap, (https://opentopomap.org accessed on 27 April 2023), and ESRI World Topo, (https:/www.esri.com accessed on 27 April 2023).

Soil samples derived from three private temperate deciduous forest stands—a young oak forest (47°18′32.40″ N; 18°49′33.60″ E), a middle-aged oak forest (47°18′43.20″ N; 18°49′51.60″ E), and a black locust forest (47°18′50.40″ N; 18°49′48.00″ E)—in the vicinity of Martonvásár and Ráckeresztúr in Central Hungary (Figure 1). The climate of the studied site is typically temperate and is referred to as the Pannonian 2 environmental zone of Europe [32]. The sampling area landscape is nearly flat, with a very slight slope to the west (1.7%). The dominant soil type is leached chernozem. The 80.6-hectare forest block containing the stands is an afforested area established in the 1930s.

The stands—which differ in age and forest structure—are within 1 km of each other. The young oak forest (T1) is in the regeneration phase (coppicing), occupies 5.71 hectares, and is dominated by pedunculate oak (*Quercus robur* L.) and Turkey oak (*Quercus cerris* L.) individuals. The forest was planted in 1933 and clear-cut in the winter of 2016. The ratio of the main tree species at harvesting was 57% (pedunculate oak) and 43% (Turkey oak). The ongoing natural reforestation is based on stump sprouts and seedlings of the former old trees, resulting in a species ratio similar to the former stand. In the soil sampling year, the forest was an open area with an average height of 1 m of saplings and sprouts (canopy closure was equal to zero). The middle-aged oak forest (T2) is an 82-year-old mixed stand with pedunculate oak (40%), Turkey oak (20%), and South European flowering ash (*Fraxinus ornus* L., 40%). According to forestry records, the forest is 5.04 hectares with a 92% canopy closure, and the basal area in order of pedunculate oak, European flowering ash, and Turkey oak are 8.3, 10.9, and 5.3 m^2^ ha^−1^. The third sampling site (A1) is a 3.58-hectare, twice-coppiced black locust forest stand (*Robinia pseudoacacia* L.). The stand contains 17-year-old trees and some sporadically located 67-year-old individuals, with 4.6 and 0.8 m^2^ ha^−1^ basal areas, respectively. The black locust forest has an 84% canopy closure (Supplementary Table S1).

### 2.2. Soil Sampling and Processing

Homogenous and typical parts of the forests were chosen for experimental purposes, where study plots of 100 m^2^ quadrates (10 × 10 m) were marked (one plot per stand was signed to secure the slightest disturbance in the forests). Soil samples were collected five times in the study plots in 2018, on 31 March, 25 May, 27 June, 27 August, and 17 October. During the sampling procedures, one-kilogram soil samples were collected on three randomly chosen points of plots involving two topsoil depths: 0–10 cm (layer A) and 10–40 cm (layer B) at each sampling time. All former sampling places were marked with wooden stakes to avoid resampling. The spade was sterilized with 80% ethanol before each sample pit excavation. Soil samples were collected from intact soil along the side wall of the pit using sterilized spatulas. All samples were stored in disposable plastic bags and kept under low-temperature conditions (4 °C) until processing. Eighteen samples per sampling time (three plots × three sampling points × two layers) were used for physical, chemical, and catabolic activity analyses. Furthermore, six composite samples were created by carefully blending soil samples derived from the same layers per plot to perform DNA isolation and metagenome analyses (three plots × two layers).

### 2.3. Soil Physical and Chemical Analyses

After roots and other visible plant residues were removed, the soil samples were air dried, ground, and sieved (mesh size 0.63 mm) for physical and chemical analyses. The fresh soil samples were weighed and subsequently dried at 105 °C until weight stability for soil moisture determination. pH_KCl_ (1 M KCl) and ${\mathrm{pH}}_{H_{2}O}$ were ascertained at a soil–solvent ratio of 1:2.5 (*w*/*v*) with a pH meter using a glass electrode [33]. Soil organic carbon (C_org_), plant available phosphorous (AL-P_2_O_5_), and nitrate nitrogen (NO_3_-N) content were determined spectrophotometrically using a Shimadzu UV-1601 spectrophotometer (Shimadzu, Kyoto, Japan) following the chromic acid oxidation method [34], ammonium lactate, and potassium chloride extraction [35]. The calcium carbonate (CaCO_3_) content was determined using a Scheibler-type calcimeter [36]. The available potassium (AL-K_2_O), calcium (AL-Ca), magnesium (AL-Mg), and sodium (AL-Na) contents were determined following ammonium lactate (AL) extraction [35] using Agilent 5110 ICP-OES analyzer (Agilent Technologies, Mulgrave, VIC, Australia). The soil textures were analyzed using the pipetting method following 0.5 M sodium pyrophosphate (Na_4_P_2_O_7_) treatment [37]. The total nitrogen (TN) content of the samples was determined by dry combustion [38] using a CN628 analyzer (LECO, St. Joseph, MI, USA); the total carbon (TC), total organic (TOC), and total inorganic carbon (TIC) contents were analyzed by dry combustion [39] using an RC612 analyzer (LECO, St. Joseph, MI, USA).

### 2.4. DNA Extraction and High-Throughput Sequencing

Community DNA was extracted from 0.25 g of soil using the DNeasy PowerSoil Kit (Qiagen, Hilden, Germany) following the manufacturer’s instructions. The V3-V4 region of the 16S rRNA gene was amplified using universal bacteria-specific primers (Bakt_341F: 5′-CCTACGGGNGGCWGCAG-3′ and Bakt_805R: 5′-GACTACNVGGGTATCTAATCC-3′) [40] with Illumina adapter overhang sequences added to their 5′ ends. All composite soil samples were amplified in triplicates to preclude inter-sample variability. The amplicon–PCR reaction mix contained 7.5 µL of 2× Phusion Flash PCR Master mix (Thermo Fischer Scientific, Vilnius, Lithuania), 3 µL of each primer (1 µM) (Merck, Feltham, UK), and 1.5 µL of template DNA. The PCR involved an initial denaturation at 95 °C for 3 min, 25 cycles of denaturation at 95 °C for 30 s, annealing at 55 °C for 30 s, elongation at 72 °C for 30 s, and a final extension at 72 °C for 5 min. The plates were stored at 4 °C in the PCR machine after completion of the program. Agencourt AMPure XP beads (Beckman Coulter, Indianapolis, IN, USA) were used to purify the PCR products. Amplification was checked with a Qiagen QIAxcel Advanced System (Qiagen, Hilden, Germany) using a DNA Screening cartridge (Qiagen, Hilden, Germany). Triplicate PCR products from the same samples were pooled and mixed in one tube. Amplicons were indexed with a second PCR according to the manufacturer’s instructions using dual indices of the Illumina Nextera XT Index Kit. The libraries were subsequently normalized using Qubit 4.0 fluorometer (Invitrogen, Thermo Fischer Scientific, Singapore), then pooled in equimolar ratios and sequenced using the Illumina MiSeq platform (Illumina, San Diego, CA, USA) with the MiSeq Reagent Kit v2 (2 × 250 bp) run configuration in the Centre for Agricultural Research at the Agricultural Institute in Martonvásár, Hungary. The sequencing reaction was prepared according to the Illumina 16S metagenomic protocol.

The raw 16S rRNA gene sequencing data are available at the NCBI Sequence Read Archive (SRA) (http://www.ncbi.nlm.nih.gov/sra accessed on 31 January 2023) under accession number PRJNA929690 [SAMN32968865-32968894].

### 2.5. Bioinformatical Data Analysis

In total, 8,847,952 paired-end short reads (an average of 33,674 per sample) were generated. From these, 4,423,976 contigs were constructed and processed in multiple steps using mothur v1.47 [41] following the MiSeq SOP pipeline (www.mothur.org accessed on 20 January 2023). First, the sequence reads were screened for ambiguous bases, sequence length (minlength = 400 and maxlength = 500), and homopolymers, resulting in 2,644,459 sequences, of which 1,999,155 were unique.

Then, the sequences were aligned to the ARB-SILVA SSU Ref NR 138 reference database (http://www.arb-silva.de/ accessed on 20 January 2023) using the default settings and the Needleman-Wunsch pairwise alignment method [42]. The aligned sequences were filtered for gap characters (terminal and vertical) and clustered based on their abundance using the pseudo-single linkage algorithm developed by Huse et al. [43], resulting in 2,521,683 sequences. The vsearch program (included with the mothur installer) was used to detect chimeric sequences [44]. Chimera removal resulted in 1,627,208 sequences. Extremely rare sequences (singletons) were removed by filtering for abundance, thus further reducing the number to 1,117,481 [45]. Abundant sequences were classified using the Wang approach, with a minimum bootstrap confidence score set to 80% [46]. Lastly, undesirable OTUs (operational taxonomic units) other than bacteria were excluded from further downstream analyses.

The OptiClust method was employed to assign sample sequences to OTUs [47]. For this, a distance matrix was constructed between all unique sequences, keeping only sequences with more than three counts in the entire dataset [45]. Then, sequences were clustered using the default settings (cutoff = 0.03). The final sequence set used for diversity statistics consisted of 1,116,120 sequences.

The number of each OTU was counted in each sample, and their relative abundance was calculated using mothur. Diversity indices—including observed OTUs (Sobs), Chao1, Ace, inverse Simpson, and Shannon—were calculated to the smallest data set (n = 9879) using mothur. Based on the OTU composition, bacterial community structure was established by NMDS (nonmetric multidimensional scaling) using the Bray–Curtis distance matrix as implemented in mothur. NMDS scores for each axis were processed further in R Statistical Software version 4.2.0 [48].

### 2.6. MicroResp Substrate-Induced Catabolic Activity Measurements

The MicroResp™ system was used to evaluate the catabolic activity pattern of soil sample bacterial communities. This technique is based on the colorimetric detection of CO_2_ evolved from the soil after adding carbon substrates [49]. Twenty-three different carbon sources and ultrapure distilled water as a control were used in four replicates distributed into adequate 96-well plates. The following substrates were applied: L-glutamic acid (Glu), L-3,4-dihydroxy-benzoic acid (Dhb), and L-arginine (Arg) in 12 mg mL^−1^; L-asparagine-monohydrate (Asp) and L-glutamine (Gln) in 20 mg mL^−1^; DL-malic acid (Mal), Na-succinate (Suc), citric acid (Cit), D-gluconic-acid-potassium salt (Gla), L-lysine (Lys), L-alanine (Ala), and L-serine (Ser) in 40 mg mL^−1^; L-arabinose (Ara), D-xylose (Xyl), D-galactose (Gal), D-glucose (Glc), D-fructose (Fru), L-rhamnose (Rha), D-mannose (Man), trehalose (Tre), Myo-inositol (Ino), D-mannitol (Mat), and D-sorbitol (Sor) in an 80 mg mL^−1^ concentration. The pH of the substrate solutions was adjusted to 6.5 by 1 M NaOH or 1 M HCl. The plates were read at the beginning and after six hours of incubation. Indicator color changes were photometrically detected using an Anthos 2010 photometer (Biochrom, Cambridge, UK) at 570 nm. Then, the substrate-induced respiration rates were calculated from the normalized CO_2_ data after the incubation period. Finally, respiration data (μg CO_2_-C g soil^−1^ h^−1^) were standardized by the average respiration rate for each plate.

### 2.7. Data Preparation, Illustration, and Descriptive Statistics

The basic statistics of respiration, diversity, and relative abundance values retrieved from the catabolic activity and genetic surveys detailed above were executed using R [48]. The normality of the distribution of all datasets was tested with the Shapiro–Wilk normality test [50,51]. Diversity indices, bacterial relative abundance and respiration data, Kruskal–Wallis rank sum test [52], and pairwise Wilcoxon rank sum test [53] were performed using the package ‘stats’ version 4.2.0 in R to reveal and pairwise test the significant differences between stands on OTU numbers [48]. Between-group differences were analyzed using a linear model fitted on the dataset using the ‘stats’ package. The estimated marginal means (least-squares means) were calculated to illustrate significant differences, and compact letter displays were created with the packages ‘emmeans’ version 1.8.6 [54] and ‘multcomp’ version 1.4-23 [55]. The descriptive statistics of the respiration values were calculated using the ‘pastecs’ package version 1.3.21 [56] in R. The Pearson correlation coefficient [57] was calculated using the “cor” order in package ‘stats’ in R to test the influence of soil chemical parameters on the relative abundance of bacterial taxa. The correlation analysis was complemented with redundancy analysis (RDA) using the package ‘vegan’ version 2.6-2 [58] and ‘packfor’ version 0.0-8 [59] in R. RDA was also performed on environmental and standardized respiration datasets to reveal which soil parameters affect respiration activity most. The differences in substrate-induced respiration patterns of the forest stands were evaluated by nonmetric multidimensional scaling analysis [60] (NMDS) using the package ‘vegan’. The ‘ggplot2’ version 3.3.6 [61] and ComplexHeatmap version 2.13.2 [62] packages were used in R for data visualization. The figures were edited using the image and photo editing software paint.net version 5.0.13 [63]. The maps illustrating sampling sites were created with QGIS software version 3.28.5-1 [64].

## 3. Results

### 3.1. Physical and Chemical Properties of the Forest Soils

The most remarkable difference between the soils appeared in their lime content. Both layers of the T1 stand were calcareous (5.41 and 10.6%), while among the mature stands, only the deeper layer of the A1 stand contained lime in a small amount (0.92%; Table 1). The values of several chemical parameters, like TIC and AL-Ca content and pH, reflected the lime content differences of the soils. Concerning these parameters, all values of the young oak stand were remarkably higher than the elder forest stands. As for TOC, the black locust stand showed the highest value in layer A (3.10%) and the middle-aged oak stand in layer B (2.06%).

As expected, the black locust stand showed the highest TN and NO_3_-N content in layers A and B (TN = 0.26 and 0.16%, NO_3_-N = 11.50 and 4.98 mg kg^−1^ in layers A and B, respectively). As for AL-K_2_O, the black locust soil samples were rich in this compound (layer A: 32.9 mg 100 g^−1^; layer B: 29.8 mg 100 g^−1^).

### 3.2. Bacterial Community Diversity

Altogether, 1,116,120 bacterial sequences (from 9879 to 79,519 per sample) were obtained and clustered into 12,859 OTUs (Supplementary Figure S1 illustrates the rarefaction curves). Considering the OTU-derived diversity indices (S_obs_, ACE, Chao1, inverse Simpson, Shannon), all showed increasing values in both soil layers in the following order: middle-aged oak forest, young oak forest, and black locust forest. In layer A, only S_obs_, inverse Simpson, and Shannon values showed significant differences among the forest stands (S_obs_: A1–T1; inverse Simpson: A1–T2, T1–T2; Shannon: A1–T2), while in layer B, all diversity values of A1 were significantly higher than those of the oak stands. The bacterial community diversity of the young and middle-aged oak forests did not differ significantly in any index in layer B (Supplementary Figures S2 and S3).

### 3.3. Bacterial Community Structure of Forest Soils

**Figure 2:**
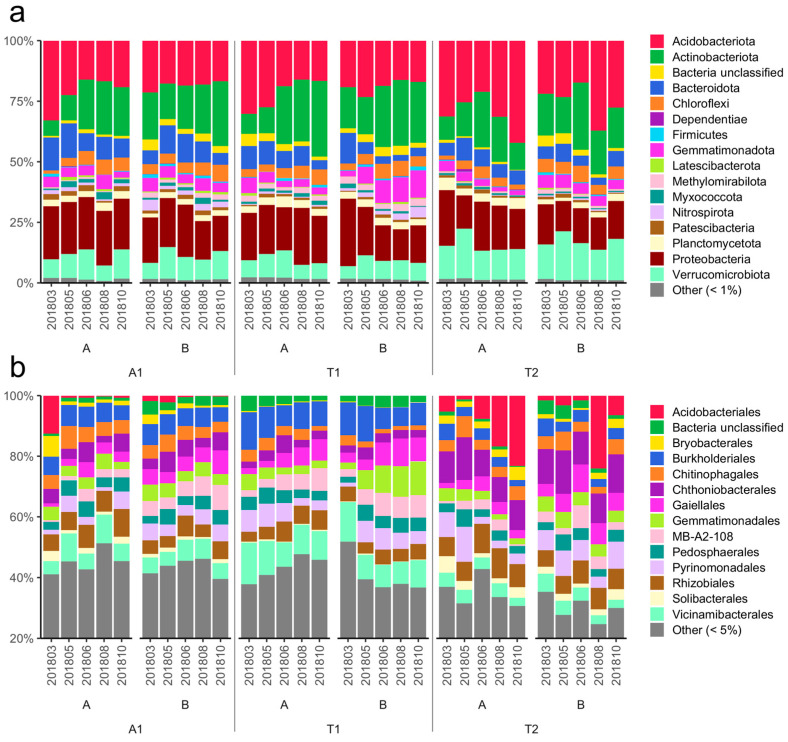
Relative abundances of major bacterial taxa at phylum (**a**) and order (**b**) levels in the investigated forest soils. Relative abundance is expressed as the percentage of total sequences. Taxa under 1% (phylum) and 5% (order) are summarized and marked as Other (<1%) and Other (<5%), respectively. Abbreviations: A1: black locust forest; T1: young oak forest; T2: middle-aged oak forest; A: 0–10 cm soil layer; B: 10–40 cm soil layer; sampling date: (yyyymm). Section 2.2 presents the exact soil sampling dates.

The relative abundance values of bacterial taxa at phylum and order levels were used to characterize the soil bacterial communities of the three investigated forest stands. Considering the whole dataset, the six primary bacterial phyla that dominated the bacterial communities were Acidobacteriota, Actinobacteriota, Proteobacteria, Verrucomicrobiota, Bacteroidota, and Gemmatimonadota, with average relative abundances of 22.8%, 18.6%, 18.5%, 10.9%, 7.3%, and 4.7%, respectively (Figure 2). In layer A, Bacteroidota, Gemmatimonadota, and Proteobacteria showed the highest relative abundance in A1; Actinobacteriota in T1; and Acidobacteriota and Verrucomicrobiota in T2. However, significant differences in relative abundances were revealed only in the case of Bacteroidota (A1–T2) and Verrucomicrobiota (T1–T2). In layer B, the most abundant phyla in A1 were Bacteroidota and Proteobacteria; in T1 Actinobacteriota and Gemmatimonadota; and in T2 Acidobacteriota and Verrucomicrobiota. Significant differences between the stands in relative abundance were detected only in the case of Verrucomicrobiota (A1–T2, T1–T2; Supplementary Figure S4).

The bacterial communities were highly diverse at the order level. Out of the 322 taxa revealed, the six most dominant orders were Vicinamibacterales, Chthoniobacterales, Burkholderiales, Rhizobiales, Pyrinomonadales, and Gaiellales, with average relative abundances of 6.7%, 6.3%, 6.3%, 6.1%, 5.1%, and 4.8%, respectively (Figure 2). Vicinamibacterales and Burkholderiales exhibited the highest relative abundance in T1 in both layers when comparing the forest stands, while Pyrinomonadales, Rhizobiales, and Chthoniobacterales were highly represented in the T2 stand in both layers. Furthermore, the highest relative abundance of Gaiellales was detected in T1 in the upper layer and T2 in the deeper layer (Supplementary Figure S5). 

**Figure 3:**
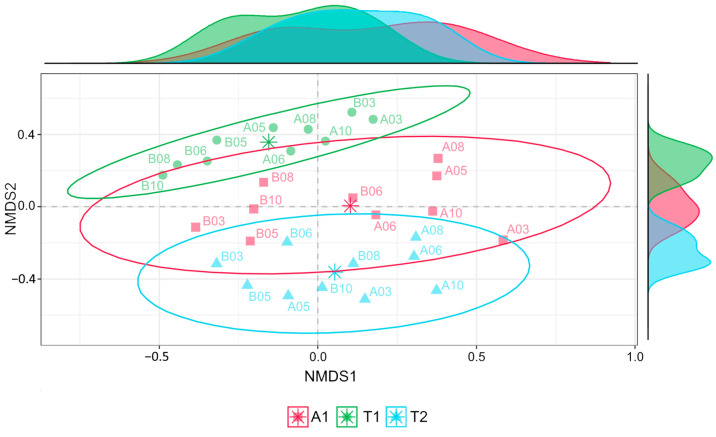
Non-metric multidimensional scaling (NMDS) diagram. Results are based on dissimilarities in the composition of bacterial communities expressed on the OTU level. Density curves indicate the distribution of points along the NMDS axes; centroids are marked with asterisks. Stress = 0.11. Abbreviations: A1: black locust forest; T1: young oak forest; T2: middle-aged oak forest; A: 0–10 cm soil layer; B: 10–40 cm soil layer; sampling date: (mm).

Figure 3 illustrates the location of the samples by their OTU-derived dimension scores in a two-dimensional NMDS diagram plane (stress = 0.11). Point cloud locations indicate that the soil bacterial community structure of the two oak stands differ remarkably. In contrast, the difference in bacterial community composition between the oak stands and the black locust forest is much lower.

### 3.4. Relationship between Bacterial Communities and Forest Soil Properties

According to the Pearson correlation coefficients, the relative abundance of Acidobacteriota and Verrucomicrobiota correlated significantly negatively with pH and TC; Proteobacteria and Bacteroidota significantly positively with TOC and C_org_; and Actinobacteriota and Gemmatimonadota significantly positively with pH and TIC (the heatmap representing the connections between phylum relative abundances and edaphic parameters is illustrated in Supplementary Figure S6). 

**Figure 4:**
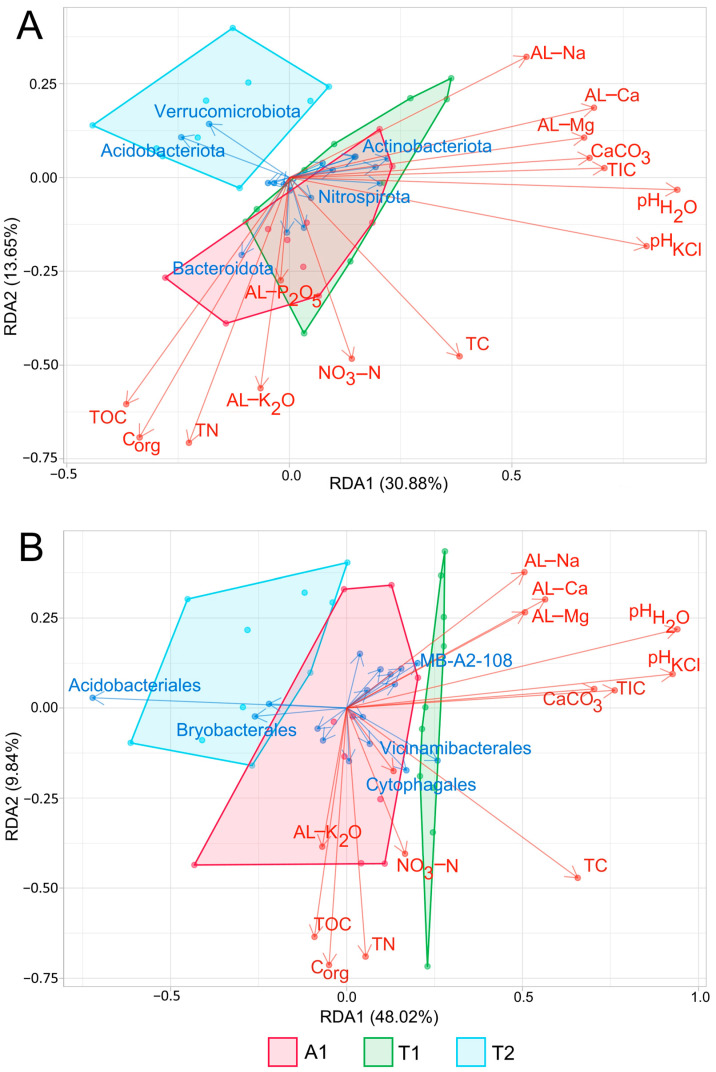
Redundancy Analysis (RDA) correlation biplot of phylum (**A**) and order (**B**) relative abundances and soil parameter values. Arrow length refers to the extent of the contribution of variables to the total variance. The angle between the arrows corresponds to the correlation between variables (an angle of 90° represents zero correlation, while an angle of 0° or 180° represents maximal positive or negative correlation, respectively). The biplot illustrates the first five taxa (blue letters), disposing of the highest variation among the samples. Polygons mark the site scores of the different stands. Abbreviations: A1: black locust forest, T1: young oak forest, T2: middle-aged oak forest. Table 1 lists the physicochemical parameter abbreviations.

As RDA analysis revealed, pH, C_org_, and TN considerably affected the inter-stand variability of the bacterial communities at the phylum level, while pH, TC, TIC, and C_org_ at the order level (Figure 4). According to the forward selection analysis, ${\mathrm{pH}}_{H_{2}O}$ was the best explanatory variable that influenced the variance of soil bacterial composition at both taxonomic levels (phylum: r^2^ = 0.24, *p* = 0.001; order: r^2^ = 0.44, *p* = 0.001). Considering the effect of the different taxa on inter-stand variability, the role of some primary taxa, namely Acidobacteriota, Bacteroidota, Verrucomicrobiota, and Actinobacteriota, was found to be dominant at the phylum level to a nearly similar extent. However, at the order level, Acidobacteriales proved to be the most dominant taxa in determining inter-stand variability.

### 3.5. Catabolic Activity Profiles of Soil Bacterial Communities

**Figure 5:**
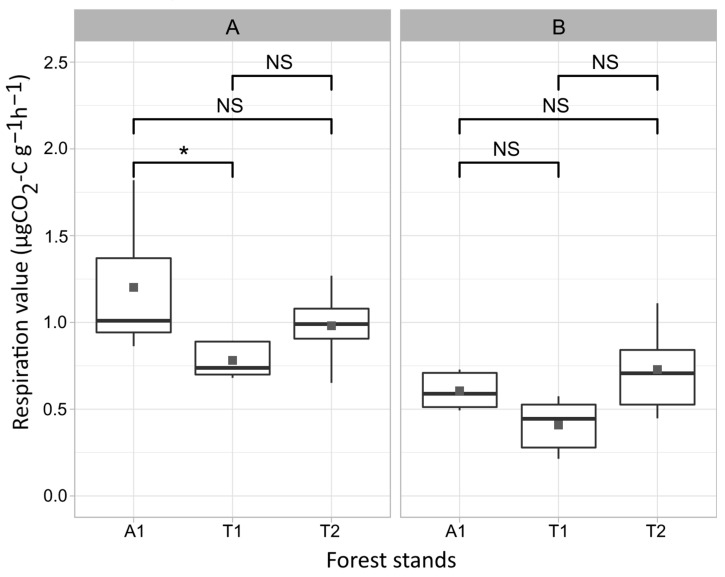
Average substrate-induced respiration values of the different forest stands. The diagrams represent the average values counted by the layers separately (**A**,**B**). Asterisks indicate significant differences at a significance level of 0.01 < *p* ≤ 0.05 (*), while NS means no significant differences. Thick horizontal lines sign the median, while dark grey dots show the mean values. Abbreviations: A1: black locust forest, T1: young oak forest, T2: middle-aged oak forest, A: 0–10 cm soil layer, B: 10–40 cm soil layer.

**Figure 6:**
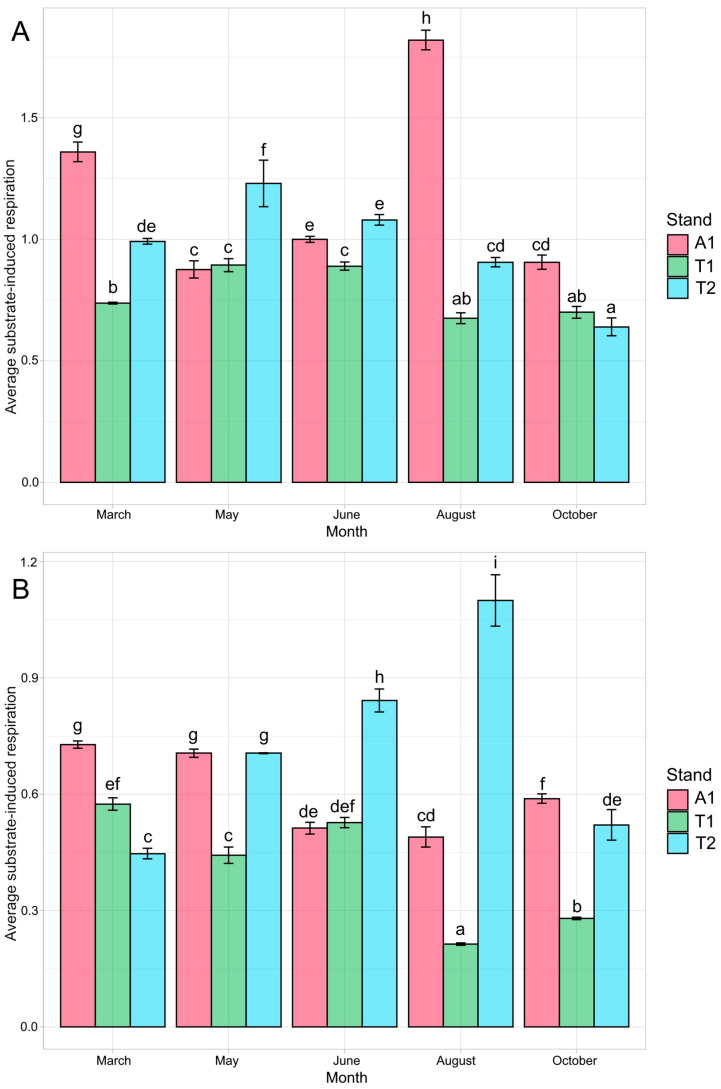
Monthly average substrate-induced respirations of the forest stands in layers A (**A**) and B (**B**). Letters correspond to the homologous groups; similar letters demonstrate no significant differences. Abbreviations: A1: black locust forest; T1: young oak forest; T2: middle-aged oak forest; A: 0–10 cm soil layer; B: 10–40 cm soil layer.

According to the substrate-induced respiration values, the bacterial communities in the older stands showed higher activity than the young oak forest; in the upper layer, the black locust forest, while in the deeper layer, the middle-aged oak forest soil samples had the highest average respiration. That is, the forest in the regeneration phase showed the lowest respiration activity in both layers in 2018 (Figure 5; Supplementary Table S2 summarizes average respiration values). In layer A, the black locust stand reached the highest substrate-induced respiration in August, and the young oak forest and the middle-aged oak forest in May. In the deeper layer, the black locust and the young oak forest reached the highest respiration in March, while the middle-aged oak forest in August (Figure 6). According to the average respiration values, the most exploited carbon sources were Mal, Glc, Suc, Fru, and Asp in soil layer A, while those in soil layer B were Mal, Glc, Fru, Suc, and Cit (Supplementary Table S2).

**Figure 7:**
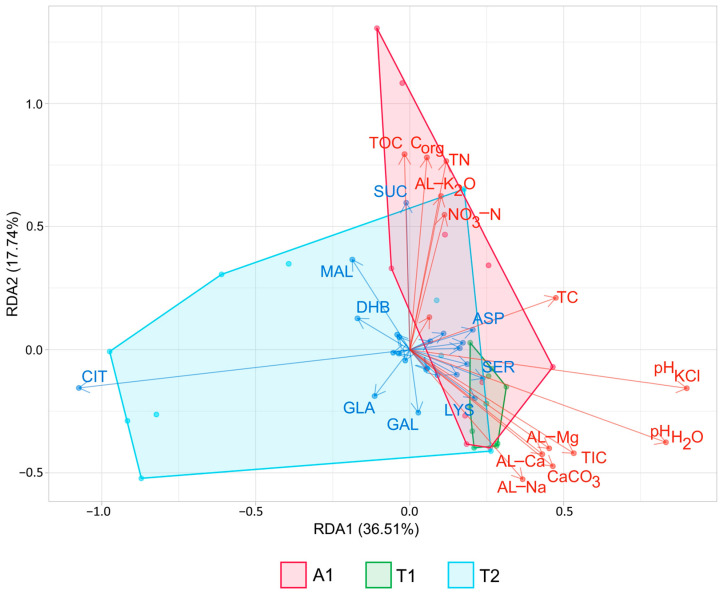
Redundancy analysis (RDA) correlation biplot of carbon source utilization and soil parameter values. Arrow length refers to the extent of the contribution of variables to the total variance. The angle between arrows corresponds to the correlation between variables (an angle of 90° represents zero correlation, while an angle of 0° or 180° represents maximal positive or negative correlation, respectively). The biplot illustrates the first nine carbon sources (blue letters), disposing of the highest variation among the samples. Polygons represent the site scores of the different stands. Abbreviations: A1: black locust forest; T1: young oak forest; T2: middle-aged oak forest. Table 1 contains the abbreviations of physicochemical parameters. The Materials and Methods chapter includes the abbreviations of carbon sources.

As the results of the RDA analysis highlighted, Cit had the highest effect among the substrates investigated on the variance of respiration. Considering this carboxylic acid, the T2 stand revealed the highest utilization; the difference in Cit consumption compared to the other stands was remarkable in both layers (Supplementary Figure S7). According to the RDA results, Cit consumption correlated negatively with pH and TC. Furthermore, Mal and Suc were also determinant substrates on between-stand variability. Both compounds were utilized in higher amounts in the upper layer of the older stands, and primarily, Suc showed a strong positive relationship with soil nutrient content (TOC, C_org_, TN, AL-K_2_O, NO_3_-N). The forward selection analysis also highlighted the significance of acidity and humus content, which marked pH_KCl_ and C_org_ as good predictors of respiration patterns (r^2^ = 0.31 and 0.13 at *p* = 0.01, respectively). As the RDA and the NMDS analyses revealed, there were no substantial differences in substrate respiration patterns between the young oak forest and the two other stands (Figure 7 and Supplementary Figure S8). However, the two older stands showed a remarkable difference in respiration pattern. Based on the two figures, it is assumable that the alteration in respiration can be traced back primarily to the differences in Cit consumption in the summer and autumn periods. 

## 4. Discussion

According to the bacterial diversity of the soil layers investigated, our results found that the two oak stands were more similar to the black locust stands than to each other. The causes of the considerable diversity alteration between the oak forests can lie in forest structural and ecological differences of the stands. As is already known, clearcutting (which also occurred in the young oak forest two years before sampling) significantly affects soil bacterial community composition and diversity [30,31]. However, it is noteworthy that the direction of the difference in our study was the opposite of what was expected. Namely, the bacterial diversity of the soil in the young oak stand proved to be higher than that of the middle-aged oak forest. Former studies’ results suggest two different possible change directions. First, the higher diversity may be attributable to the short time since the clearcutting event [30]. This assumption posits that the initial diversity of the bacterial microbiome was somewhat higher in the young oak forest and that not enough time has passed to show a firm change. However, it is also conceivable that the abundance of some rare taxa increased rapidly after the harvest [65], which altered the diversity in favor of the young oak forest stand.

On the other hand, other, primarily edaphic parameters, could also be responsible for the firm differences in bacterial diversity between the oak stands. As Fierer and Jackson [66] highlighted, soil pH best predicts soil bacterial richness and diversity, and bacterial diversity is higher at neutral than acidic pH. The latter statement corresponds well to our results because the soil under the young oak forest was neutral (layer A) and slightly basic (layer B), whereas under the middle-aged oak it was acidic in both layers.

Edaphic parameters, mainly pH, carbon and nutrient content, largely influence soil bacterial composition and functioning [22,67,68,69]. Our results denoted the pH values and C_org_ and TN content as the main drivers of the variability of bacterial community structure, where Acidobacteriota, Actinobacteriota, Verrucomicrobiota, and Bacteroidota formed the primary source of inter-stand variance. Our analyses on the soil parameter–phylum relative abundance connections revealed a strong connection between Acidobacteriota and soil pH; the detected strong negative correlation was expected since bacteria belonging to some subdivisions of this phylum prefer soil conditions with low pH [70,71]. In their comparative study, Fierer et al. [72] detected that the relative abundance of Acidobacteriota negatively correlates with the C mineralization rate (CMR). Although we did not calculate this parameter, considering that CMR has a strong positive correlation with TOC [73], our results were not in correspondence with the results of Fierer et al. (Acidobacteriota did not show a correlation with TOC at all; Pearson r = 0.04), suggesting an environment-dependent behavior of Acidobacteriota. 

Although Dong et al. [74] and Lan et al. [75] reported an opposite connection between the phyla Actinobacteriota and Gemmatimonadota and soil organic carbon content and pH, these phyla behaved similarly in our study. The relative abundances of both phyla correlated negatively with TOC and C_org_ and positively with pH. In this context, the behavior of Actinobacteriota and Gemmatimonadota seemed to be oligotrophs in the semi-arid site we investigated. Similarly, Li et al. [76] also found a negative correlation between the relative abundances of these phyla and TOC. Overall, this kind of independence from organic carbon (and the positive correlation with inorganic carbon) suggests a different metabolic profile for the bacteria that allows them to utilize nutrient-poor environments (in their comparative study, Li et al. [76] found the highest relative abundance of Gemmatimonadota in desert soils).

Bacteria belonging to the phylum Verrucomicrobiota can dominate habitats where the quality and quantity of organic matter restrict the growth of copiotrophic bacteria [77]. In this respect, the strong negative correlation between Verrucomicrobiota and TC is reasonable, even though Ranjan et al. [78] found very different results when investigating the Amazon rainforest. Based on this, it is likely that under constrained nutrient availability (e.g., in semi-arid chernozem soils), the lower competitiveness of Verrucomicrobiota is more emphasized against copiotroph bacteria. The abundance of the latter group is more dependent on available C sources, a finding our results also support. 

As Fierer et al. [72] detected, the relative abundances of beta-Proteobacteria and Bacteroidota positively correlate with the C mineralization rate (C availability), indicating their copiotroph-like behavior. These findings are reflected in the positive correlation between the relative abundance of Proteobacteria and Bacteroidota and C sources (TC, C_org_, and TOC) revealed by us. Furthermore, the alterations experienced in their relative abundances compared to oligotroph bacteria (Acidobacteriota and Verrucomicrobiota) also refer to their labile C source-dependent growth. However, as Fierer et al. [72] noted, not all member taxa of these phyla are distinctly copiotrophic.

Many studies have reported the significant effect of phosphorous on soil microbiome composition [22,79,80,81], which our results on RDA could not confirm, at least not directly; phosphorous was the least diversifying factor at both taxonomic levels. However, it should be noted that the lowest AL-P_2_O_5_ content was measured under the middle-aged oak forest in both layers (the difference compared to the other stands was greater in the upper layer), where Acidobacteriales showed the highest relative abundance, especially in August and October. The bacterial orders whose relative abundance correlates negatively with soil phosphorus content can play a vital role in the element cycle by making phosphorus bioavailable [82]. In this sense, the members of Acidobacteriales were likely to participate in meeting the elevated phosphorus needs of the mature oak forest, which were raised by physiological processes that functioned in the late vegetation period. The assumption of the elevated need for phosphorus is supported by the high citrate consumption in the T2 stand soil observed. Indeed, citrate efflux in certain circumstances enhances phosphorus mobilization and uptake by plants [83,84].

Although the orders of symbiotic nitrogen-fixing bacteria (Burkholderiales and Rhizobiales) belonged to well-represented taxa in the black locust forest, their relative abundance was not outstanding and did not reach the values shown in the oak forests. In contrast to our results, Xu et al. [85] revealed a remarkable change in soil bacterial composition decades after black locust afforestation on the Loess Plateau, China, where the bacterial community composition shifted towards the dominance of nitrogen-fixing taxa. However, the decades-long nitrogen-fixing activity was likely to considerably increase the nitrate content of the black locust soil investigated in our study, which could negatively impact this activity and, later, the relative abundance of bacterial taxa involved in the nitrogen fixation process. The observation that the available external nitrogen sources mitigate the rate of nitrogen fixation of free-living diazotrophs supports this [86]. Although the total nitrogen content of the collected soil samples was much lower (3.5×), the nitrate content considerably exceeded (3.7×) the values observed by Xu et al. [85], which is likely to support this concept.

Concerning the differences in respiration activity between the forest stands, we revealed a closer similarity in the microrespiration patterns between the soil samples of black locust and middle-aged oak stands than in the young oak forest (the latter stand showed the lowest substrate-induced respiration in both layers). According to our hypothesis, the lower respiration activity of the topsoil under the young oak stand is caused by the altered physical conditions of the soil that appeared after the clearcutting of the former forest stand. Due to the lack of a closed canopy, more solar radiation reaches the soil surface, and the area is more exposed to wind, which facilitates the desiccation of the upper soil layers. The results of Brockett et al. [87] and Xu et al. [85] support this hypothesis, proving that the soil moisture content significantly impacts the functional potential of the soil microbiome. Furthermore, the different pH values of the forest soils investigated reinforce the assumption. Namely, even though the young oak forest soil samples had higher pH values, their substrate-induced respiration was lower than the closed stands’. Indeed, through the investigation of 53 mature broad-leaved forests, Bååth and Anderson [88] revealed that the substrate-induced respiration of topsoil samples correlates positively with soil pH. It suggests that the harvest of the former forest triggered a lower level of microbial respiration.

The causes of the considerable increase in soil respiration activity in the black locust (layer A) and the middle-aged oak (layer B) stand soil samples in August remain unclear. Higher nutrient content (TOC, TN, NO_3_-N, AL-P_2_O_5_, AL-K_2_O) in layer A is one possible explanation for the highest respiration in the black locust stand, which, together with rain events, could elevate the respiration activity [89]. As for the middle-aged oak stand, periodic shoot development, typical for pedunculate oak [90,91], could be a reason for the phenomenon. Based on their root respiration investigation on beech (*Fagus sylvatica* L.), Epron et al. [92] confirmed the positive relationship between root respiration and root and shoot development. The rate of root respiration and the root exudate flux are positively linked in oaks [93]. The latter process can directly affect bacterial population sizes along roots [94], which can cause a periodic rise in respiration activity. Of course, finding the causes of respiration elevation experienced in the two stands requires more detailed and long-term investigations.

The strong relationship between soil characteristics—mainly the soil pH and organic carbon content—and the soil respiration activity is well known [95,96,97,98]. Our results have also strengthened this observation. At the same time, our results highlight the importance of the differences in forest types in influencing soil bacterial composition and activity; however, as Thoms et al. and Urbanová et al. suggest, this occurs mainly in an indirect way, implying that the effect of trees appears in the modification of soil parameters, which affects the bacterial community composition and functioning [99,100]. Thoms et al. denote that this indirect effect can switch to a direct influence seasonally [99]. Further studies are needed to confirm our results on the seasonal increase in bacterial respiration in the rhizosphere of the middle-aged oak stand and its relationship with the periodic shoot development of pedunculate oak. Notwithstanding, our results seem to underpin Thoms et al., namely that in addition to the indirect effect of trees, a seasonal, direct impact influencing the soil microbial functioning also exists [99].

We had no opportunity to investigate the physicochemical and microbial attributes that characterized the soils before the afforestation because of the lack of control areas where traditional agricultural techniques were used over the past 90 years. Therefore, the exact direction and extent of the change in soils caused by land use change were not determinable. However, according to history maps and forest inventory data, it is apparent that the three forest stands were established on the former arable land at nearly the same time and thus influenced the soil’s microbial processes over several decades of their growth. Therefore, the differences in soil bacterial community structure and diversity between the two closed forests (black locust and middle-aged oak stands) on similar soils (slightly acidic leached chernozems) derive mainly from the differences in the main tree species. Similar to other studies [25,99], our results proved the tree species’ effect on soil microbial community structure and diversity. Although our research did not aim to examine the ramifications of forest harvesting on soil microbial functioning, our results allowed us to determine the direction of microbial activity change that occurred by forest disturbance indirectly. Namely, compared with the other stands, the lowest substrate-induced respiration and the highest pH values suggest a respiration decrease after clearcutting in the young oak forest. This statement is consistent with other studies, which revealed the negative impact of forest harvest on soil microbial respiration [28,30]. Overall, our results bring valuable information to a deeper understanding of the connection between the forest types and their soil bacterial communities.

## 5. Conclusions

Different types of forests established under similar ecological circumstances showed differences in soil bacterial community structure and respiration activity. Among the soil physicochemical parameters, soil pH had the most considerable effect on soil bacterial community structure, emphasizing that the initial soil conditions (in our case, the limestone content) remarkably influence the soil bacterial community. Considering that the bacterial community diversity of the slightly disturbed semi-natural middle-aged oak forest was lower than the twice-coppiced non-indigenous forestry plantation (black locust), our results proved that closer-to-nature forest management does not necessarily induce more complex soil bacterial microbiomes. The recently clearcut forest did not indicate lower soil bacterial diversity but possessed a substantially weaker activity than the mature stands. Thus, our results suggest an adverse effect of this type of forest harvesting on soil microbial life.

## Figures and Tables

**Table 1:** Average soil physical and chemical properties of the samples collected in the forest stands from March to October 2018.

Layer	Stand	TC	TOC	TIC	C_org_	${\mathrm{pH}}_{H_{2}O}$	pH_KCl_	CaCO_3_
		(%)	(%)	(%)	(%)			(%)
A	A1	**3.43 ± 0.82 ^a^**	**3.10 ± 0.72 ^a^**	**0.33 ± 0.12 ^ab^**	**4.40 ± 0.72 ^a^**	**6.29 ± 0.57 ^a^**	**6.09 ± 0.72 ^a^**	**0.00 ± 0.00 ^a^**
	T1	**3.65 ± 0.19 ^a^**	**2.44 ± 0.13 ^ab^**	**1.17 ± 0.19 ^a^**	**3.89 ± 0.25 ^a^**	**7.17 ± 0.09 ^b^**	**6.93 ± 0.06 ^b^**	**5.41 ± 1.15 ^b^**
	T2	**2.38 ± 0.25 ^b^**	**2.22 ± 0.20 ^b^**	**0.17 ± 0.06 ^b^**	**3.32 ± 0.24 ^b^**	**5.82 ± 0.60 ^a^**	**5.14 ± 1.10 ^a^**	**0.00 ± 0.00 ^a^**
B	A1	**2.39 ± 0.34 ^a^**	**1.97 ± 0.16 ^ab^**	**0.42 ± 0.26 ^ab^**	**3.01 ± 0.23 ^a^**	**6.97 ± 0.27 ^ab^**	**6.62 ± 0.18 ^ab^**	**0.92 ± 1.33 ^a^**
	T1	**3.45 ± 0.34 ^b^**	**1.62 ± 0.14 ^a^**	**1.81 ± 0.44 ^a^**	**2.44 ± 0.20 ^b^**	**7.54 ± 0.16 ^a^**	**7.11 ± 0.03 ^a^**	**10.60 ± 2.39 ^b^**
	T2	**2.22 ± 0.15 ^a^**	**2.06 ± 0.09 ^b^**	**0.20 ± 0.08 ^b^**	**2.95 ± 0.13 ^a^**	**6.37 ± 0.41 ^b^**	**5.88 ± 0.73 ^b^**	**0.00 ± 0.00 ^a^**
Layer	Stand	TN	NO_3_-N	AL-P_2_ O_5_	AL-K_2_ O	AL-Na	AL-Mg	AL-Ca
		(%)	(mg kg^−1^)	(mg 100 g^−1^)	(mg 100 g^−1^)	(mg kg^−1^)	(mg g^−1^)	(mg g^−1^)
A	A1	**0.26 ± 0.06 ^a^**	11.50 ± 16.50 ^a^	11.40 ± 3.08 ^a^	**32.90 ± 7.71 ^a^**	40.60 ± 13.60 ^a^	0.62 ± 0.24 ^a^	**5.43 ± 5.06 ^ab^**
	T1	**0.20 ± 0.01 ^ab^**	2.81 ± 2.53 ^a^	10.40 ± 3.21 ^a^	**21.80 ± 1.86 ^b^**	80.40 ± 21.70 ^a^	1.05 ± 0.53 ^a^	**25.00 ± 19.70 ^a^**
	T2	**0.16 ± 0.02 ^b^**	1.29 ± 1.94 ^a^	8.74 ± 2.18 ^a^	**22.90 ± 2.32 ^b^**	42.60 ± 9.10 ^a^	0.51 ± 0.06 ^a^	**2.43 ± 0.74 ^b^**
B	A1	**0.16 ± 0.02 ^a^**	4.98 ± 6.06 ^a^	8.50 ± 3.63 ^a^	**29.80 ± 5.96 ^a^**	47.40 ± 15.20 ^a^	**0.82 ± 0.35 ^ab^**	**12.80 ± 10.40 ^ab^**
	T1	**0.13 ± 0.01 ^b^**	0.75 ± 1.30 ^a^	8.72 ± 3.30 ^a^	**16.60 ± 2.15 ^b^**	83.20 ± 22.00 ^a^	**1.22 ± 0.41 ^a^**	**34.50 ± 20.10 ^a^**
	T2	**0.15 ± 0.01 ^ab^**	0.09 ± 0.19 ^a^	8.41 ± 3.47 ^a^	**22.10 ± 1.19 ^ab^**	50.80 ± 7.16 ^a^	**0.48 ± 0.04 ^b^**	**3.09 ± 0.81 ^b^**

Abbreviations: Layer, A: 0–10 cm, B: 10–40 cm; Stand: forest stands; A1: black locust forest; T1: young oak forest; T2: middle-aged oak forest; TC: total carbon; TOC: total organic carbon; TIC: total inorganic carbon; C_org_: organic carbon; CaCO_3_: calcium carbonate; TN: total nitrogen; NO_3_-N: nitrate nitrogen; AL-P_2_O_5_: ammonium lactate soluble phosphorous; AL-K_2_O: ammonium lactate soluble potassium; AL-Na: ammonium lactate soluble sodium; AL-Mg: ammonium lactate soluble magnesium; AL-Ca: ammonium lactate soluble calcium. Significant differences at *p* = 0.05 are marked with bold characters, while homologous groups with the letters a and b.

## Data Availability

The raw 16S rRNA gene sequencing data are available at the NCBI Sequence Read Archive (SRA) (http://www.ncbi.nlm.nih.gov/sra accessed on 31 January 2023) under accession number PRJNA929690 [SAMN32968865-32968894].

## Supplementary Materials

The following supporting information can be downloaded at: https://www.mdpi.com/article/10.3390/microorganisms12061162/s1, Figure S1: Rarefaction curves of the different samples based on 16S rRNA gene amplicon sequencing. Abbreviations: A1: black locust forest, T1: young oak forest, T2: middle-aged oak forest; A: 0–10 cm soil layer, B: 10–40 cm soil layer; sampling date: (yyyymm). Section 2.2 presents the exact soil sampling dates; Figure S2: The mean number of OTUs revealed in soil samples of the different forest stands. Asterisks indicate significant differences at a significance level of 0.01 < *p* ≤ 0.05 (*) and 0.001 < *p* ≤ 0.01 (**), while NS means no significant differences. Abbreviations: A1: black locust forest, T1: young oak forest, T2: middle-aged oak forest; A: 0–10 cm soil layer, B: 10–40 cm soil layer; Figure S3: Mean diversity index values of the different forest soil bacterium communities. Asterisks indicate significant differences at a significance level of 0.01 < *p* ≤ 0.05 (*) and 0.001 < *p* ≤ 0.01 (**), while NS means no significant differences. Abbreviations: A1: black locust forest, T1: young oak forest, T2: middle-aged oak forest; A: 0–10 cm soil layer, B: 10–40 cm soil layer; Figure S4: Comparison of forest soils by average relative abundance values of the six primary phyla in the upper (A) and deeper (B) layers. Results of the Pairwise Wilcoxon Rank Sum Test are indicated above the upper line: asterisks indicate significant differences at a level of 0.01 < *p* ≤ 0.05 (*) and 0.001 < *p* ≤ 0.01 (**), while NS means no significant differences. Abbreviations: A1: black locust forest, T1: young oak forest, T2: middle-aged oak forest, A: 0–10 cm soil layer, B: 10–40 cm soil layer. In boxplots, thick black lines represent medians, while dark grey squares represent mean values; Figure S5: Comparison of forest soils by average relative abundance values of the six primary order in the upper (A) and deeper (B) layers. Results of the Pairwise Wilcoxon Rank Sum Test are indicated above the upper line: asterisks indicate significant differences at a level of 0.01 < *p* ≤ 0.05 (*) and 0.001 < *p* ≤ 0.01 (**), while NS means no significant differences. Abbreviations: A1: black locust forest, T1: young oak forest, T2: middle-aged oak forest, A: 0–10 cm soil layer, B: 10–40 cm soil layer. In boxplots, thick black lines represent medians, while dark grey squares represent mean values; Figure S6: Pearson correlation heatmap of the relative abundance values of bacterial phyla and different soil physicochemical parameters. The colour palette refers to the value of the Pearson correlation coefficient (r). Asterisks indicate significant differences at a significance level of 0.01 < *p* ≤ 0.05 (*), 0.001 < *p* ≤ 0.01 (**), *p* ≤ 0.001 (***), while empty cells sign no significant differences. Taxa under 1% are summarized and marked as other (<1%). Abbreviations of physicochemical parameters are shown in Table 1; Figure S7: Average utilization values of the six primary substrates in the upper (A) and deeper (B) layers of the forest stands investigated. Results of the Pairwise Wilcoxon Rank Sum Test are indicated above the upper line: asterisks indicate significant differences at a level of 0.01 < *p* ≤ 0.05 (*) and 0.001 < *p* ≤ 0.01 (**), while NS means no significant differences. Abbreviations: A1: black locust forest, T1: young oak forest, T2: middle-aged oak forest, A: 0–10 cm soil layer, B: 10–40 cm soil layer. In boxplots, thick black lines represent medians, while dark grey squares represent mean values. Abbreviations of carbon sources are shown in the Materials and Methods chapter. Figure S8: Nonmetric multidimensional scaling (NMDS) diagram on substrate utilization patterns of the forest stands investigated. The results are based on dissimilarities in the substrate utilization of bacterial communities. Density curves indicate the 95% probability level. Abbreviations: T1: T1 oak forest, T2: T2 oak forest; A: 0–10 cm soil layer, B: 10–40 cm soil layer; Table S1: Main characteristics of the studied forest stands; Table S2: Average carbon source utilization (respiration) values of forest soil layers expressed in µgCO_2_-C g^-1^ h^-1^. Numbers in brackets represent standard errors marked with italic characters. Abbreviations: A1: black locust forest, T1: young oak forest, T2: middle-aged oak forest, A: 0–10 cm soil layer, B: 10–40 cm soil layer, Avg.: average values. Abbreviations of carbon sources are shown in the Materials and Methods chapter.
