# Supplementary material for: Soil Parameters and Forest Structure Commonly Form the Microbiome Composition and Activity of Topsoil Layers in Planted Forests
Source: Microorganisms. 2024 Jun 6;12(6):1162. doi: 10.3390/microorganisms12061162 (PMC11205539; doi:10.3390/microorganisms12061162)
Supplement: Supplementary file 1 [file microorganisms-12-01162-s001.zip › microorganisms-3029085-supplementary.pdf]

Supplementary Tables

Table S1: Main characteristics of the studied forest stands.

Forest type	Year of establishment	Year of harvest	Vegetation composition (%)	Basal area (m^2 ha^{-1})	Site extension (ha)
Young oak forest (T1)	1933	2016	*Quercus robur* 57%<br>*Quercus cerris* 43%	-	5.71
Middle-aged oak forest (T2)	1936	-	*Quercus robur* 40%<br>*Quercus cerris* 20%<br>*Fraxinus ornus* 40%	8.3<br>5.3<br>10.9	5.04
Black locust forest (A1)	1933	1962 and 1992	*Robinia pseudoacacia* 100%	5.4	3.58

Table S2: Average carbon source utilization (respiration) values of forest soil layers expressed in μgCO_2-C g^{-1} h^{-1}. Numbers in brackets represent standard errors marked with italic characters. Abbreviations: A1: black locust forest, T1: young oak forest, T2: middle-aged oak forest, A: 0–10 cm soil layer, B: 10–40 cm soil layer, Avg.: average values. Abbreviations of carbon sources are shown in the Materials and Methods chapter.

	A1<br>A	A1<br>B	T1<br>A	T1<br>B	T2<br>A	T2<br>B	Means<br>A	Means<br>B
Gal	0.90 *(0.37)*	0.61 *(0.13)*	0.82 *(0.11)*	0.42 *(0.15)*	0.88 *(0.17)*	0.73 *(0.27)*	0.86 *(0.23)*	0.59 *(0.22)*
Tre	1.09 *(0.52)*	0.49 *(0.10)*	0.76 *(0.18)*	0.34 *(0.13)*	0.88 *(0.18)*	0.67 *(0.30)*	0.91 *(0.34)*	0.50 *(0.23)*
Ara	1.32 *(0.57)*	0.66 *(0.12)*	0.88 *(0.11)*	0.44 *(0.19)*	1.01 *(0.24)*	0.79 *(0.22)*	1.07 *(0.39)*	0.63 *(0.22)*
Glc	1.73 *(0.80)*	0.77 *(0.13)*	1.19 *(0.21)*	0.54 *(0.21)*	1.39 *(0.38)*	0.87 *(0.24)*	1.44 *(0.54)*	0.73 *(0.24)*
Fru	1.54 *(0.63)*	0.76 *(0.13)*	1.06 *(0.15)*	0.50 *(0.20)*	1.15 *(0.26)*	0.86 *(0.34)*	1.25 *(0.43)*	0.71 *(0.27)*
Xyl	1.34 *(0.49)*	0.71 *(0.10)*	0.99 *(0.09)*	0.48 *(0.20)*	1.04 *(0.22)*	0.78 *(0.22)*	1.12 *(0.33)*	0.66 *(0.21)*
Rha	0.96 *(0.18)*	0.52 *(0.10)*	0.58 *(0.08)*	0.35 *(0.14)*	0.81 *(0.15)*	0.61 *(0.23)*	0.78 *(0.21)*	0.50 *(0.19)*
Man	1.33 *(0.58)*	0.67 *(0.09)*	0.87 *(0.12)*	0.44 *(0.18)*	1.11 *(0.35)*	0.79 *(0.21)*	1.10 *(0.42)*	0.64 *(0.22)*
Cit	1.23 *(0.48)*	0.58 *(0.14)*	0.72 *(0.10)*	0.41 *(0.17)*	1.66 *(0.48)*	1.02 *(0.40)*	1.20 *(0.54)*	0.67 *(0.36)*
Mal	2.24 *(0.89)*	0.85 *(0.14)*	1.13 *(0.15)*	0.58 *(0.23)*	1.74 *(0.59)*	1.11 *(0.50)*	1.70 *(0.74)*	0.84 *(0.38)*
Suc	1.93 *(0.65)*	0.67 *(0.15)*	0.85 *(0.09)*	0.43 *(0.19)*	1.28 *(0.40)*	1.01 *(0.69)*	1.35 *(0.62)*	0.70 *(0.46)*
Dhb	1.18 *(0.39)*	0.48 *(0.11)*	0.56 *(0.09)*	0.31 *(0.10)*	0.93 *(0.20)*	0.64 *(0.23)*	0.89 *(0.36)*	0.48 *(0.20)*
Ino	0.97 *(0.43)*	0.50 *(0.08)*	0.64 *(0.13)*	0.34 *(0.13)*	0.69 *(0.22)*	0.52 *(0.18)*	0.77 *(0.30)*	0.45 *(0.15)*
Mat	0.96 *(0.17)*	0.55 *(0.11)*	0.68 *(0.10)*	0.37 *(0.13)*	0.81 *(0.15)*	0.62 *(0.21)*	0.82 *(0.18)*	0.51 *(0.18)*
Sor	1.00 *(0.31)*	0.59 *(0.11)*	0.72 *(0.09)*	0.39 *(0.15)*	0.88 *(0.27)*	0.64 *(0.20)*	0.87 *(0.25)*	0.54 *(0.18)*
Gla	0.94 *(0.22)*	0.60 *(0.13)*	0.64 *(0.12)*	0.38 *(0.13)*	0.98 *(0.24)*	0.76 *(0.23)*	0.85 *(0.24)*	0.58 *(0.22)*
Ala	0.90 *(0.26)*	0.54 *(0.12)*	0.64 *(0.10)*	0.36 *(0.14)*	0.72 *(0.29)*	0.65 *(0.33)*	0.75 *(0.24)*	0.52 *(0.24)*
Lys	0.52 *(0.15)*	0.41 *(0.11)*	0.43 *(0.07)*	0.31 *(0.12)*	0.40 *(0.11)*	0.42 *(0.18)*	0.45 *(0.12)*	0.37 *(0.14)*
Gln	1.14 *(0.36)*	0.62 *(0.20)*	0.75 *(0.11)*	0.40 *(0.16)*	0.80 *(0.15)*	0.68 *(0.33)*	0.90 *(0.28)*	0.57 *(0.24)*
Arg	0.66 *(0.20)*	0.39 *(0.09)*	0.46 *(0.09)*	0.30 *(0.11)*	0.52 *(0.18)*	0.48 *(0.20)*	0.55 *(0.18)*	0.39 *(0.15)*
Glu	1.15 *(0.31)*	0.57 *(0.12)*	0.73 *(0.10)*	0.39 *(0.14)*	0.88 *(0.13)*	0.64 *(0.25)*	0.92 *(0.26)*	0.53 *(0.20)*
Asp	1.52 *(0.63)*	0.72 *(0.10)*	1.02 *(0.10)*	0.47 *(0.17)*	1.12 *(0.53)*	0.78 *(0.28)*	1.22 *(0.50)*	0.66 *(0.23)*
Ser	1.06 *(0.26)*	0.70 *(0.13)*	0.85 *(0.09)*	0.43 *(0.16)*	0.87 *(0.16)*	0.69 *(0.32)*	0.93 *(0.20)*	0.61 *(0.24)*
Avg.	1.20 *(0.58)*	0.61 *(0.16)*	0.78 *(0.22)*	0.41 *(0.16)*	0.98 *(0.41)*	0.73 *(0.32)*	0.99 *(0.46)*	0.58 *(0.26)*
	0.90 *(0.52)*		0.59 *(0.27)*		0.85 *(0.39)*		0.78 *(0.43)*	

Supplementary Figures

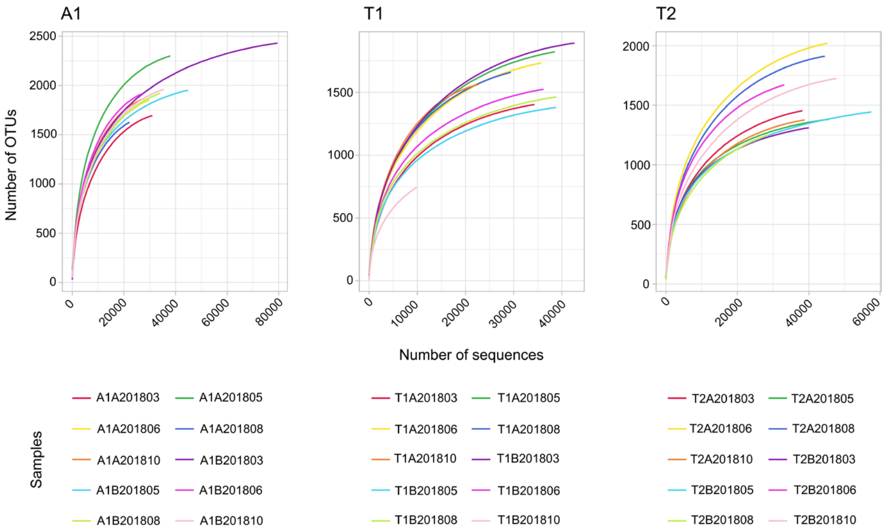

Figure S1: Rarefaction curves of the different samples based on 16S rRNA gene amplicon sequencing. Abbreviations: A1: black locust forest, T1: young oak forest, T2: middle-aged oak forest; A: 0-10 cm soil layer, B: 10-40 cm soil layer; sampling date: (yyyymm). Subsection 2.2 presents the exact soil sampling dates.

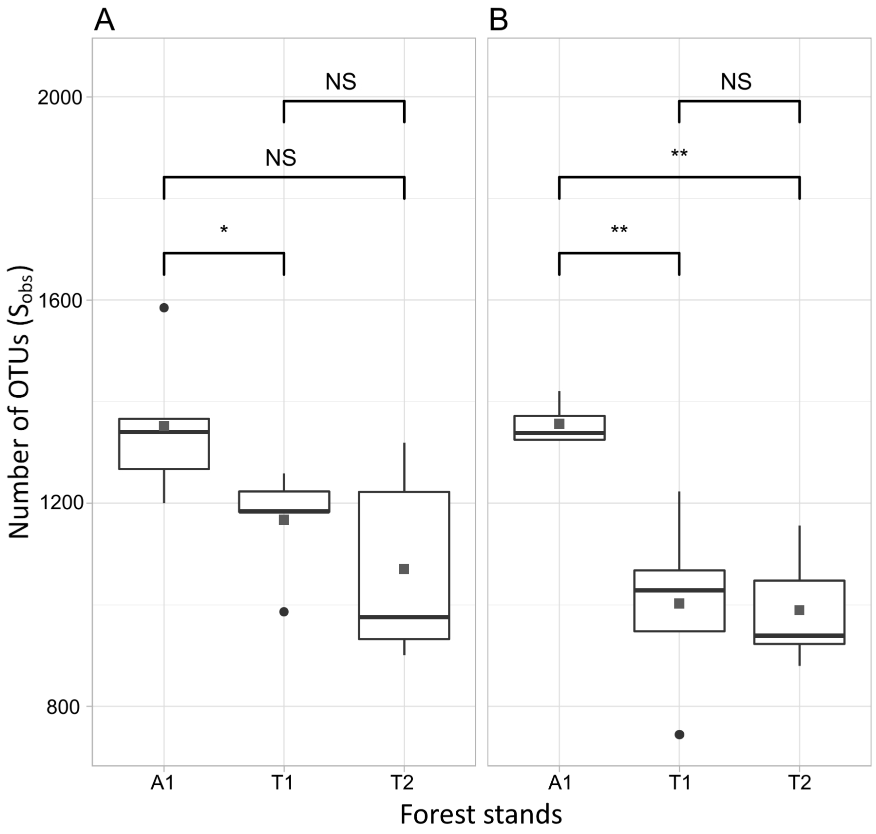

Figure S2: The mean number of OTUs revealed in soil samples of the different forest stands. Asterisks indicate significant differences at a significance level of $0.01 < p \leq 0.05$ (*) and $0.001 < p \leq 0.01$ (**), while NS means no significant differences. Abbreviations: A1: black locust forest, T1: young oak forest, T2: middle-aged oak forest; A: 0–10 cm soil layer, B: 10–40 cm soil layer.

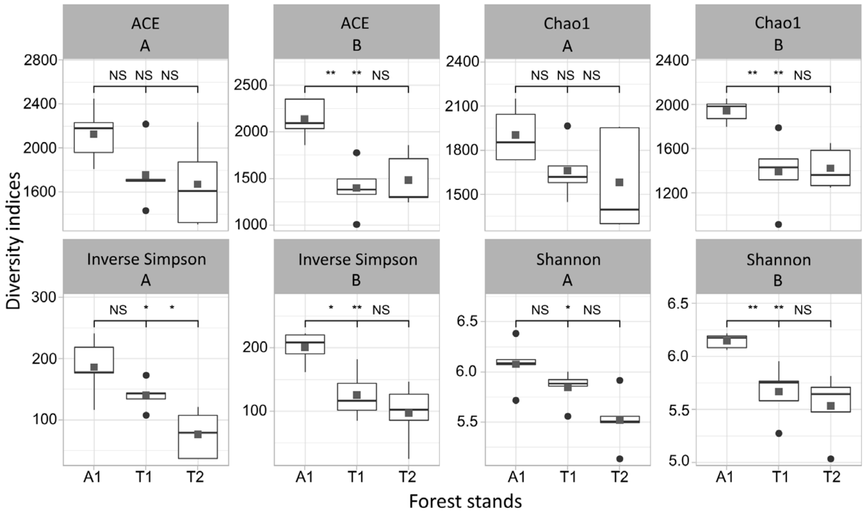

Figure S3: Mean diversity index values of the different forest soil bacterium communities. Asterisks indicate significant differences at a significance level of $0.01 < p \leq 0.05$ (*) and $0.001 < p \leq 0.01$ (**), while NS means no significant differences. Abbreviations: A1: black locust forest, T1: young oak forest, T2: middle-aged oak forest; A: 0–10 cm soil layer, B: 10–40 cm soil layer.

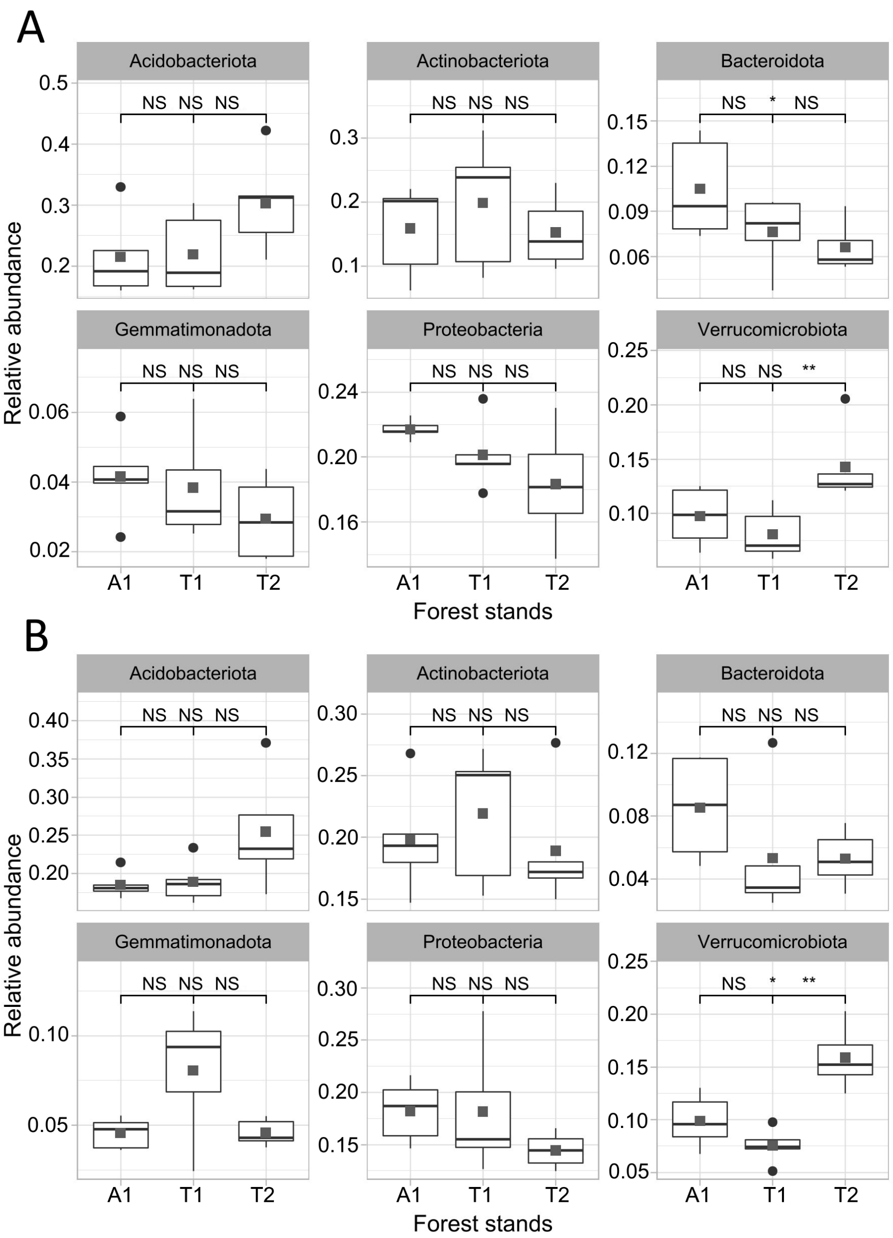

Figure S4: Comparison of forest soils by average relative abundance values of the six primary phyla in the upper (A) and deeper (B) layers. Results of the Pairwise Wilcoxon Rank Sum Test are indicated above the upper line: asterisks indicate significant differences at a level of $0.01 < p \leq 0.05$ (*) and $0.001 < p \leq 0.01$ (**), while NS means no significant differences. Abbreviations: A1: black locust forest, T1: young oak forest, T2: middle-aged oak forest, A: 0–10 cm soil layer, B: 10–40 cm soil layer. In boxplots, thick black lines represent medians, while dark grey squares represent mean values.

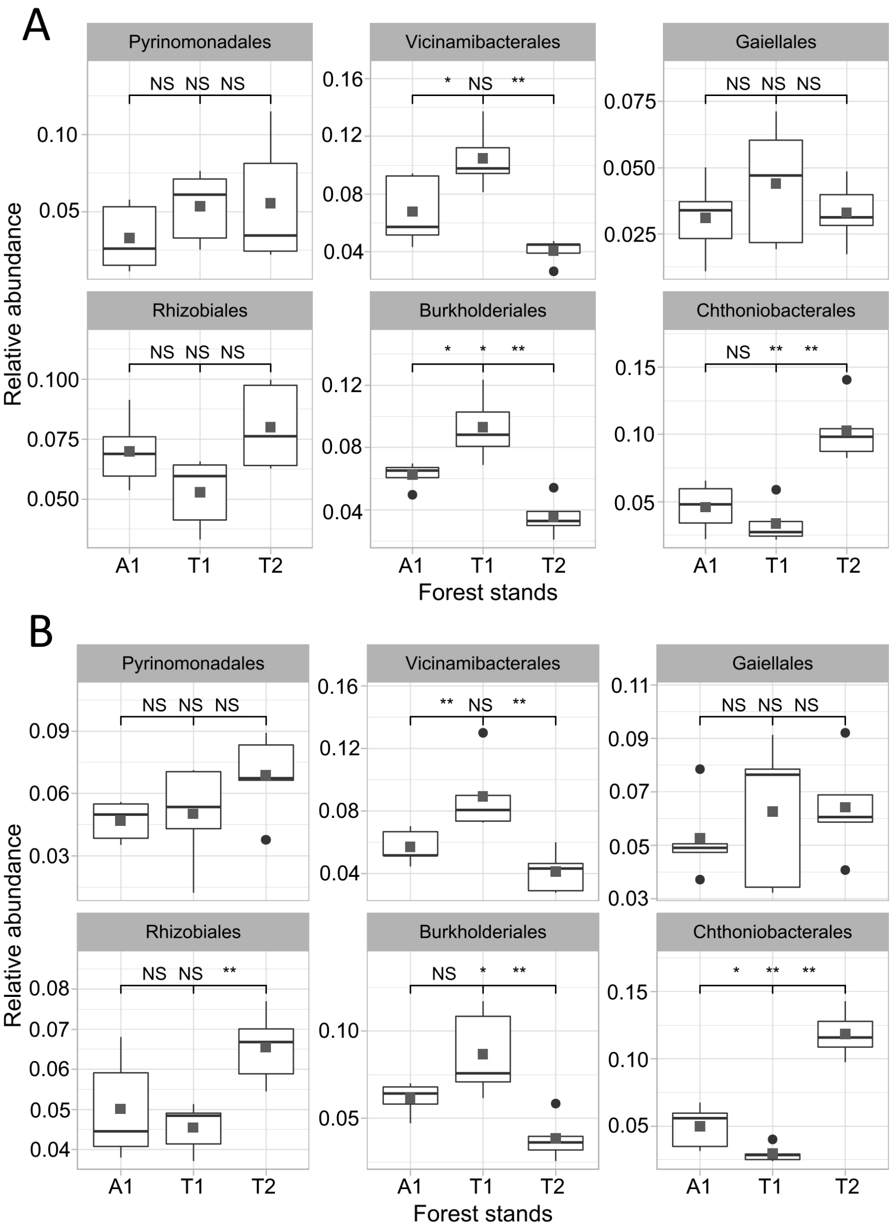

Figure S5: Comparison of forest soils by average relative abundance values of the six primary order in the upper (A) and deeper (B) layers. Results of the Pairwise Wilcoxon Rank Sum Test are indicated above the upper line: asterisks indicate significant differences at a level of $0.01 < p \leq 0.05$ (*) and $0.001 < p \leq 0.01$ (**), while NS means no significant differences. Abbreviations: A1: black locust forest, T1: young oak forest, T2: middle-aged oak forest, A: 0–10 cm soil layer, B: 10–40 cm soil layer. In boxplots, thick black lines represent medians, while dark grey squares represent mean values.

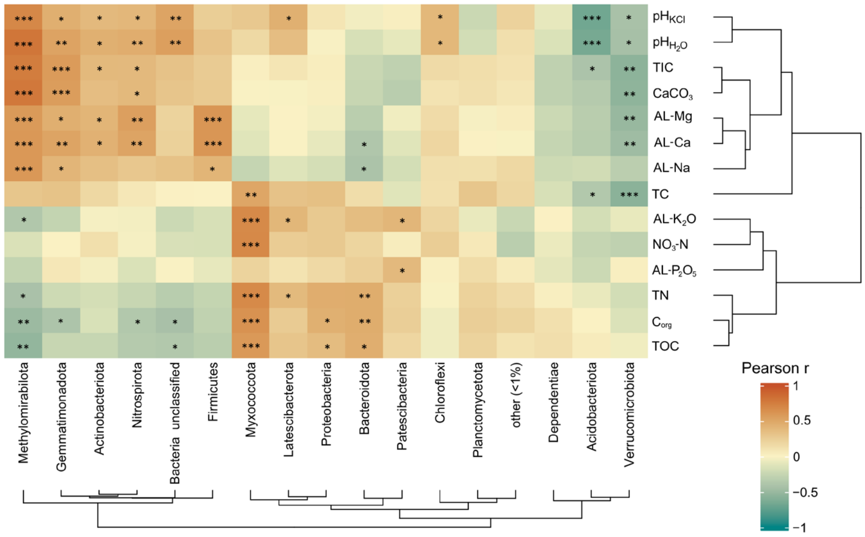

Figure S6: Pearson correlation heatmap of the relative abundance values of bacterial phyla and different soil physicochemical parameters. The colour palette refers to the value of the Pearson correlation coefficient (r). Asterisks indicate significant differences at a significance level of $0.01 < p \leq 0.05$ (*), $0.001 < p \leq 0.01$ (**), $p \leq 0.001$ (***), while empty cells sign no significant differences. Taxa under 1% are summarized and marked as other (<1%). Abbreviations of physicochemical parameters are shown in Table 1.

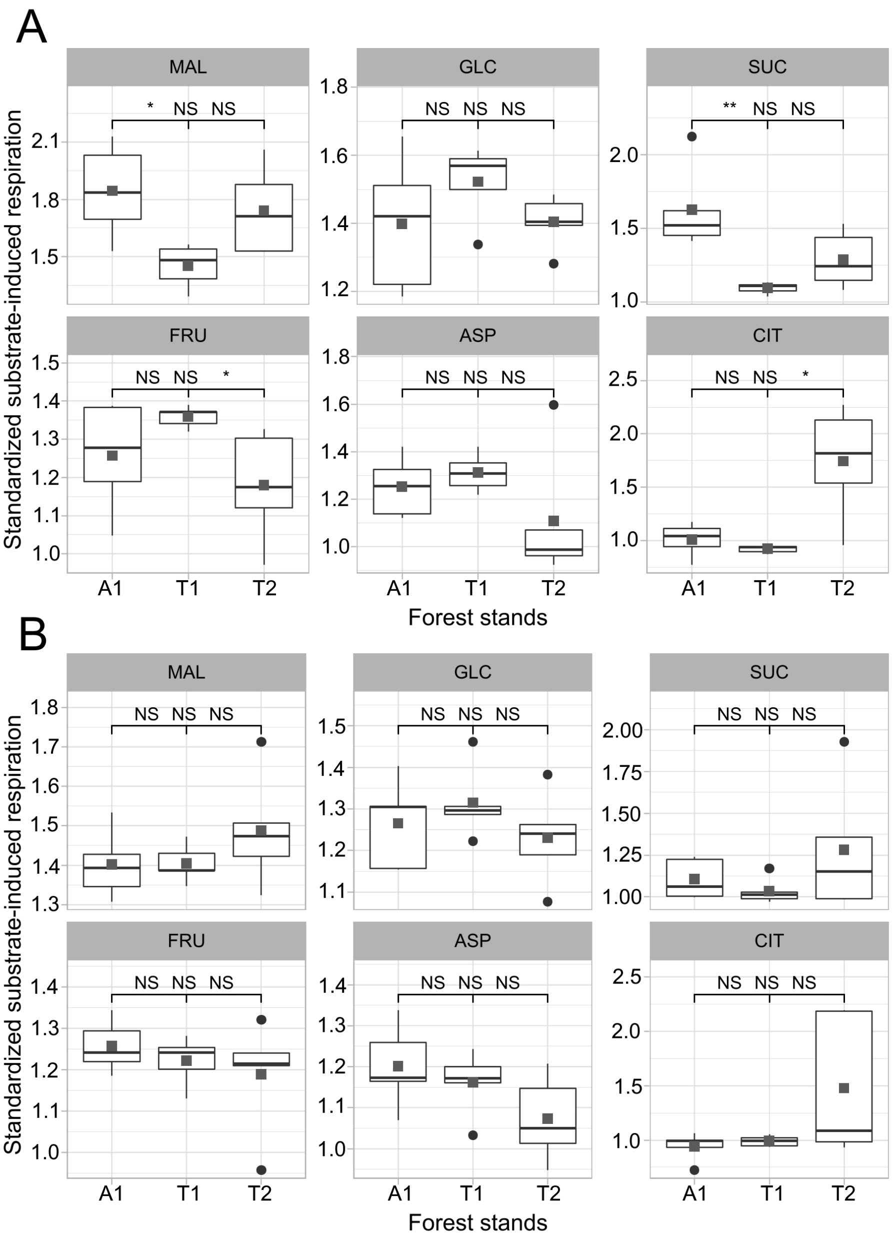

Figure S7: Average utilization values of the six primary substrates in the upper (A) and deeper (B) layers of the forest stands investigated. Results of the Pairwise Wilcoxon Rank Sum Test are indicated above the upper line: asterisks indicate significant differences at a level of $0.01 < p \leq 0.05$ (*) and $0.001 < p \leq 0.01$ (**), while NS means no significant differences. Abbreviations: A1: black locust forest, T1: young oak forest, T2: middle-aged oak forest, A: 0–10 cm soil layer, B: 10–40 cm soil layer. In boxplots, thick black lines represent medians, while dark grey squares represent mean values. Abbreviations of carbon sources are shown in the Materials and Methods chapter.

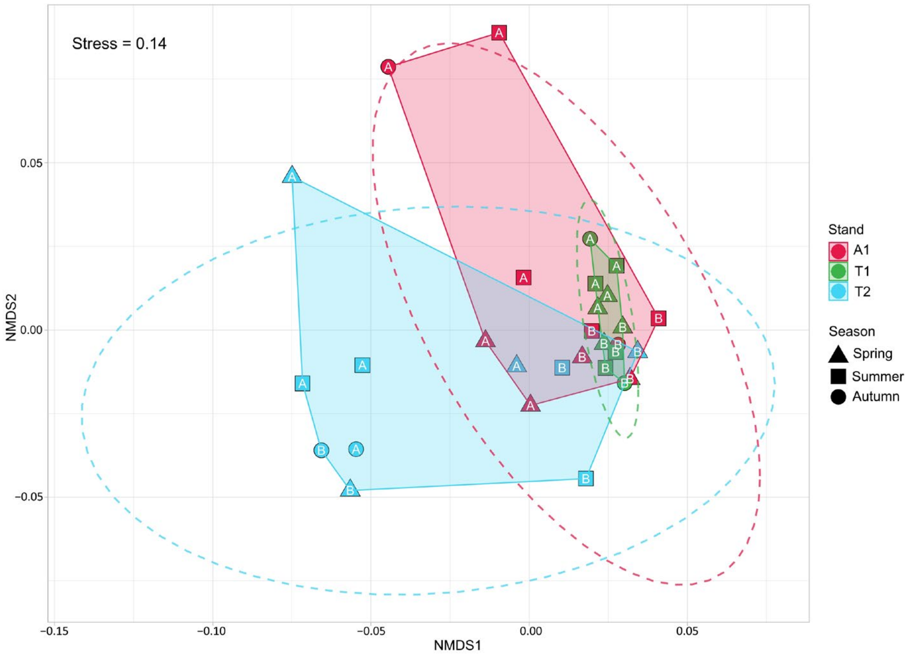

Figure S8: Nonmetric multidimensional scaling (NMDS) diagram on substrate utilization patterns of the forest stands investigated. The results are based on dissimilarities in the substrate utilization of bacterial communities. Density curves indicate the 95% probability level. Abbreviations: T1: T1 oak forest, T2: T2 oak forest; A: 0–10 cm soil layer, B: 10–40 cm soil layer.
